# Mycotoxins in Rice Correlate with Other Contaminants? A Pilot Study of the Portuguese Scenario and Human Risk Assessment

**DOI:** 10.3390/toxins15040291

**Published:** 2023-04-17

**Authors:** Liliana J. G. Silva, André M. P. T. Pereira, Sofia Duarte, Inês Pedro, Catarina Perdigão, Alexandra Silva, Celeste M. Lino, Anabela Almeida, Angelina Pena

**Affiliations:** 1 LAQV, REQUIMTE, Laboratory of Bromatology and Pharmacognosy, Faculty of Pharmacy, University of Coimbra, Polo III, Azinhaga de Sta. Comba, 3000-548 Coimbra, Portugal; 2 Department of Veterinary Sciences, Vasco da Gama Research Center, Vasco da Gama University School, 3020-210 Coimbra, Portugal; 3 CIBIT—Coimbra Institute for Biomedical Imaging and Translational Research, University of Coimbra, 3000-548 Coimbra, Portugal

**Keywords:** mycotoxins, metalloids, aflatoxin B1, ochratoxin A, zearalenone, inorganic arsenic, rice, occurrence, risk assessment

## Abstract

Rice is the second most important cereal crop and is vital for the diet of billions of people. However, its consumption can increase human exposure to chemical contaminants, namely mycotoxins and metalloids. Our goal was to evaluate the occurrence and human exposure of aflatoxin B1 (AFB1), ochratoxin A (OTA), zearalenone (ZEN), and inorganic arsenic (InAs) in 36 rice samples produced and commercialized in Portugal and evaluate their correlation. The analysis of mycotoxins involved ELISA, with limits of detection (LODs) of 0.8, 1 and 1.75 μg kg^−1^ for OTA, AFB1, and ZEN, respectively. InAs analysis was carried out by inductively coupled plasma mass spectrometry (ICP-MS; LOD = 3.3 μg kg^−1^). No sample showed contamination by OTA. AFB1 was present in 2 (4.8%) samples (1.96 and 2.20 μg kg^−1^), doubling the European maximum permitted level (MPL). Concerning ZEN, 88.89% of the rice samples presented levels above the LOD up to 14.25 µg kg^−1^ (average of 2.75 µg kg^−1^). Regarding InAs, every sample presented concentration values above the LOD up to 100.0 µg kg^−1^ (average of 35.3 µg kg^−1^), although none surpassed the MPL (200 µg kg^−1^). No correlation was observed between mycotoxins and InAs contamination. As for human exposure, only AFB1 surpassed the provisional maximum tolerable daily intake. Children were recognized as the most susceptible group.

## 1. Introduction

Rice, *Oryza sativa*, belongs to the Gramineae family and Oryzoides subfamily. It is the second most vital cereal crop worldwide [1] and an important element of the diet and subsistence of over 3.5 billion individuals [2].

According to the latest report of the European Union (EU) for the “EU agricultural outlook for markets, income and environment 2020–2030”, worldwide production of rice has been progressively increasing during the last decade. Whereas in the EU, the annual per capita consumption is 6 kg, the worldwide annual per capita consumption is 54 kg [3].

After harvest, the rice grain commonly undergoes several processing steps, such as drying, milling, and packaging, to be convenient for consumption. Rice consumption provides around 20% of the daily calories bulk for many humans [4,5] and is widely used for weaning and by infants and celiac patients due to its nutritional benefits and relatively low allergic potential [6]. The nutrient content depends on the soil variety, growth conditions, and processing [7]. The glycemic index greatly varies depending, among other things, on the type and cooking method [4]. Although brown rice presents nutritional benefits and contains more lipids, minerals, vitamins, dietary fiber, micronutrients, and bioactive compounds, polished rice is typically consumed and is an essential food, fulfilling everyday energy demands and part of the protein necessities [2].

Given the current concern about world hunger, climate change effects, growth of the population, and food security, the production of rice is endangered, along with rice quantity and quality availability [8], which might increase human exposure to chemical contaminants, such as mycotoxins or metalloids. In fact, a positive relationship was already found between rice consumption and the urinary excretion of arsenic in women from the United States [4] and mycotoxins in Swedish adolescents [9].

Among several other agricultural commodities, rice was already regarded as the greatest substrate for aflatoxin production [4]. These secondary metabolites of *Aspergillus* species are categorized by the International Agency for Research on Cancer (IARC) in group 1. In particular, aflatoxin B1 (AFB1), the most mutagenic, genotoxic, and carcinogenic mycotoxin, can originate hepatic lesions, cirrhosis, primary hepatocellular carcinoma, and Kwashiorkor and Reye’s syndrome [10]. As for ochratoxin A (OTA), human toxicity includes nephron- and hepatic toxicity, as well as teratogenicity, carcinogenicity, mutagenicity, and immunosuppression. OTA is listed in the 2B group of the IARC. OTA, produced by *Aspergillus* and *Penicillium* species, is associated with Balkan endemic nephropathy (BEN) [10]. Zearalenone (ZEN) is produced by several fungi, the most common being *Fusarium graminaerum* and *Fusarium culmorum* [10]. ZEN is not classified as carcinogenic to humans, belonging to IARC Group 3 [11]. It was proposed as more appropriate in the denomination of a nonsteroidal estrogen or a mycoestrogen [12].

Arsenic (As) abounds in the soil owing to natural and anthropogenic factors such as rock weathering, mining activities, and pesticide application [13]. It is regarded as the most toxic metalloid in rice. When a rice paddy field is flooded, in anaerobic conditions, As soil content becomes more transportable as arsenate (As (V)), which is then converted to a more bioavailable species, arsenite (As (III)). Compared to other cereal crops, larger quantities of As can be absorbed by rice roots, transferred, and accumulated into rice grains [13]. As chemical forms present substantial differences regarding toxicity. Inorganic As (InAs)—As (III) and As (V)—is the most toxic form. The ingestion of relatively low doses over a long period of time may cause organ malfunction and chronic syndromes [4], with InAs classified as a carcinogenic agent by the IARC [14].

Therefore, these toxic elements must be controlled in rice in order to meet a high standard of quality, given its massive worldwide consumption, particularly by children [15]. They are considered a vulnerable population given their lower body weight, increased metabolic rate, lower detoxification ability, and increased physiological vulnerability [16].

Current EU legislation does not establish maximum permitted limits (MPLs), specifically in rice, for either AFB1, OTA, or ZEN. However, according to the legislation in force, Regulation (EU) No. 165/2010 sets a maximum level for cereals of 2 μg kg^−1^ for individual content of AFB1 [17]. Commission Regulation (EC) No. 1881/2006 sets a maximum limit of 3 μg kg^−1^ for OTA for all products derived from unprocessed cereals, including processed cereal products and cereals intended for direct human consumption. For ZEN, the cited regulation states that “given the low contamination levels of Fusarium toxins found in rice, no maximum levels are proposed for rice or rice products”. A limit of 75 μg kg^−1^ is set for cereals intended for direct human consumption [18].

Regarding arsenic, Commission Regulation No. 2015/1006, amended Regulation No. 1881/2006 with regard to maximum levels of arsenic in inorganic form (sum of As (III) and As (V)) in foodstuffs, setting limits to the presence of inorganic arsenic in rice and rice products. A maximum of 0.2 mg kg^−1^ was set for non-parboiled milled rice (polished or white rice) and 0.25 mg kg^−1^ for parboiled and husked rice [19].

This research aimed to evaluate, for the first time, if AFB1, OTA, ZEN, and InAs co-occur and correlate in rice acquired in Portugal. Moreover, we also aimed to confirm the compliance with the maximum permitted levels of the European legislation and evaluate the exposure of the Portuguese population, more specifically children, adolescents and adults, according to their consumption of this cereal so as to assess their potential risk.

## 2. Results and Discussion

### 2.1. Frequency and Occurrence

#### 2.1.1. Mycotoxins

##### Ochratoxin A

In the present study, from the 36 samples analyzed, none showed contamination by OTA. Contamination of OTA in rice is generally lower than in wheat or corn [20].

Table 1 presents data from previous studies reported in the scientific literature on the occurrence of the selected contaminants in rice.

##### Aflatoxin B1

AFB1 was only found in 2 samples (4.8%) in concentrations of 1.96 and 2.20 μg kg^−1^, with a mean of 0.12 μg kg^−1^. Both positive samples were branded commercially acquired samples. One sample was of long wild rice, and the other was of short white rice, with their origins in Canada and Portugal, respectively. One should note that only one sample surpassed the European maximum limit of 2 μg kg^−1^.

The occurrence of aflatoxin in brown rice is often slightly higher than the MPLs established. However, after removing the husk, that content generally decreases below the maximum levels [17]. In the present study, this was not verified because the positive samples were of wild and white rice.

Comparison with other scientific studies is difficult, namely because most of the reported studies are from countries with different weather and production conditions. However, when comparing AFB1 results with previously published scientific studies, as presented in Table 1, one may observe that most published results are from the Asian continent in levels ranging from not detected to 29.8 µg kg^−1^ in Vietnam [35]. The maximum average level reported, 4.6 µg kg^−1^, regards a Pakistan study [32]. Nonetheless, high maximum levels were also reported in Austria, with 9.86 µg kg^−1^ [21] and in Canada, with 7.1 µg kg^−1^ [22]. With the exception of a Tunisian report [33], our average level was lower than those reported worldwide.

##### Zearalenone

Regarding ZEN, 88.89% (32 out of 36) of the samples presented levels above the LOD, up to 14.25 µg kg^−1^, averaging 2.75 ± 2.26 µg kg^−1^. Comparing private and white label samples, a significant difference was found (*p* = 0.0196), with white label samples showing a higher average (2.91 µg kg^−1^) than the branded ones (2.08 µg kg^−1^). Comparing brown and white rice, there was no statistical difference, but the *p*-value was near 0.05 (*p* = 0.0557), with brown samples showing a higher average. No other significant comparisons were observed.

*Fusarium* spp. cause major reductions in rice quality due to environmental conditions. High moisture and high-temperature conditions favor field *Fusarium* growth, which may also develop when rice is stored [65]. However, *Fusarium proliferatum* dominates [65], which justifies that ZEN’s natural occurrence in rice has only been scarcely reported [12]. As seen in Table 1, the average values described in the scientific literature are higher, ranging between 6.6 µg kg^−1^ in Côte d’Ivoire [51] and 143 µg kg^−1^ in Brazil [36]. However, the detection frequencies are generally lower when compared to the results of our study.

Mycotoxins are subject to regulation in many countries worldwide to limit their presence in foods. Specifically, the strict European legislation obliges the application of good practices in rice production, storage, and distribution, which may justify the current results [66]. On the other hand, the highest mycotoxin contamination values were found in the bran and husk fractions [36].

#### 2.1.2. Inorganic Arsenic

Bearing in mind the results shown in Table 2, one can perceive that all of the total samples presented inorganic rice at levels above the LOQ, up to 100.0 µg kg^−1^, with an average of 35.3 ± 28.2 µg kg^−1^. None exceeded the MPL established by the EU.

When comparing supermarket samples versus those provided by rice producers, it was found that the average contamination was higher in the latter, with 25.9 and 41.2 µg kg^−1^, respectively; a significant statistical difference was observed with a *p* = 0.0236. Comparing white and branded labels, a significant statistical difference was also observed (*p* = 0.0220), with average levels of 10.5 vs. 37.5 µg kg^−1^, respectively.

Regarding long vs. short grain, it was verified that average levels were significantly higher in the latter, with a value of 41.9 ± 28.6 µg kg^−1^ (*p* = 0.0480), up to 100.0 µg kg^−1^. Long grain had a lower average of 26.9 ± 26.1 µg kg^−1^ and a lower maximum value of 90.0 µg kg^−1^.

Accordingly, other authors (Table 1) reported that the concentrations of InAs were related to rice type and grain length. The mean content of InAs in long grains was 99 μg kg^−1^, while short grains presented a mean concentration of 122 μg kg^−1^. Therefore, short rice showed a concentration of around 30% higher As (both total As and InAs) than long rice [61]. In Finland, it was found that InAs levels in long-grain rice varied from 90 to 280 µg kg^−1^ (*n* = 8) [57], while in the UK, the grain length (long, medium, and short grains) and the InAs range were compared, and it was observed that long-grain rice ranged between 45 to 213 µg kg^−1^ [6]. On the contrary, another study compared white rice and sticky rice and verified that 2.1% of the former and 6.3% of the sticky rice contained InAs at higher concentrations than the Codex standard (0.2 mg kg^−1^); however, sticky rice showed lower mean contamination values.

The lowest *p*-value, <0.0001, was obtained when brown and white rice were compared; brown rice ranged between 23.0 and 100.0 µg kg^−1^, with an average of 55.1 ± 27.7 µg kg^−1^, while white rice ranged between >LOD and 80.0 µg kg^−1^, with an average of 22.6 ± 20.5 µg kg^−1^ (Table 2).

InAs concentrations in rice decrease in the following order: hull > bran polish > brown rice > raw rice > polished (white) rice. Therefore, commercially available polished white rice presents lower arsenic levels than whole grains and is safer for consumption [67]. In the scientific literature (Table 1), brown rice also presented increased As levels when compared to white rice (189 vs. 132 μg kg^−1^) [61]. Other authors also reported that brown rice contained a significantly higher concentration of InAs compared to white or wild rice [6]. Polishing reduces As content by removing the bran (and several nutrients), which is the fraction with the highest concentration of arsenic, followed by brown rice and white rice [58,59].

Finally, between the production regions of Tagus, Mondego, and Sado, there were no statistical differences, with average contamination values of 59.1 ± 28.0, 19.0 ± 11.3, and 32.9 ± 24.1 μg kg^−1^. Comparing rice from Portugal and abroad, it was found that Portuguese rice presented significantly higher average levels, 38.3 ± 26.1 µg kg^−1^, against 26.1 ± 33.9 µg kg^−1^ (*p* = 0.0272). According to data reported in the scientific literature, InAs levels were quite constant in temperate, subtropical, and northern hemisphere tropical regions [68].

Legislation sets maximum concentration levels of inorganic arsenic present in rice and rice derivatives [19]. Among the analyzed samples, none was found to be contaminated above the required maximum limits, which are 200 μg kg^−1^ for milled rice, 250 μg kg^−1^ for brown rice, and 100 μg kg^−1^ for rice intended for consumption by infants and young children. However, we can consider that the sample contaminated at 100 μg kg^−1^ should not be used in the diet of infants and young children. This sample was of short brown rice originating in the Tagus River provided by a rice producer.

One should note that the present study was done on uncooked rice. Pre-cooked rice presents less than 50% of the As and InAs levels of dry rice. The fact that pre-cooked rice has already been boiled leads to partial arsenic removal. It has also been stated that washing the rice before cooking or boiling it with abundant water may decrease arsenic concentrations by up to 60% [61].

#### 2.1.3. Co-Occurrence and Correlation of Mycotoxins and Inorganic Arsenic

The co-occurrence of many kinds of mycotoxins/contaminants may increase the risk to human health [69]. Two branded samples showed the co-occurrence of AFB1, ZEN, and InAs, while four (two branded and two producers’ samples) were not contaminated with any of these compounds. Nonetheless, 30 out of 36 samples (83%) presented both ZEN and InAs, a mycoestrogen and a carcinogenic agent, respectively. Nonetheless, no correlation was found between mycotoxin and InAs contamination.

### 2.2. Estimated Daily Intake and Risk Assessment

#### 2.2.1. Mycotoxins

##### Aflatoxin B1

For children, adolescent, and adult populations, different EDI assessments were carried out, bearing in mind different scenarios, including the LB approach, the UB approach (EFSA, 2010), and both combined, with the average and 95th percentile consumption [70]. For every population in the study, the maximum EDI value was 5.79 ng kg^−1^ b.w./day, calculated for children when the UB–95th consumption scenario was considered. Viewing the LB–average consumption scenario, the EDI values were 0.26 ng kg^−1^ b.w./day for children, 0.18 ng kg^−1^ b.w./day for adolescents, and 0.12 ng kg^−1^ b.w./day for adults.

**Figure 1:**
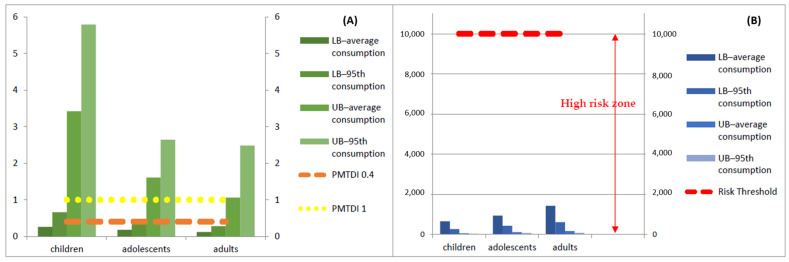
AFB1 estimated daily intake (EDI) and risk assessment: (**A**) PMTDI approach; (**B**) MOE approach.

Risk assessment was achieved through the comparison of the obtained EDI with the Kuiper–Goodman PMTDI values [71], 1.0 ng kg^−1^ b.w./day, for adults and children without hepatitis B, and 0.4 ng kg^−1^ b.w./day for those with hepatitis B virus. As seen in Table 3, children presented the higher risk values, following adolescents and adults. The percentage of EDI/PMTDI 1.0, considering the LB-average consumption scenario, was 26.2% for children, 18.2% for adolescents, and 12.0% for adults. This percentage increased when the 95th percentile was taken into account. Unsurprisingly, when considering the lower PMTDI value, the calculated risk rose significantly, up to 1447.6%, for children with the UB–95th consumption. In Figure 1A, one can more easily observe the comparison between the calculated EDI and the PMTDI of 1 and 0.4 ng kg^−1^ b.w./day. The PMTDI of 1 ng kg^−1^ b.w./day was only surpassed when the UB approaches were considered. As for the lower PMTDI of 0.4 ng kg^−1^ b.w./day, even the LB approach with 95th percentile consumption of children surpassed it.

Regarding the MOE approach, and considering that a value inferior to 10,000 represents a concern for human health [72], one can perceive, both in Figure 1B and Table 3, that the calculated risk for every scenario and population group is well lower than this threshold, with maximum MOE values of 1420.8 for the adult population at the LB–average consumption, indicating high risk for every scenario considered.

According to a Pakistani study [30], the mean exposure to AFB1 through rice consumption corresponded to 22.2 ng kg^−1^ b.w./day, a value much higher than the EDI obtained in the present study.

One should note that the exposure and risk evaluation of the present study were estimated in uncooked rice. Previous studies reported that cooking or food processing can significantly reduce mycotoxin levels, including AFB1 [53]. Specifically, an average decrease of around 45.0% was observed for AFB1 present in rice as a result of washing and cooking [15].

As mentioned, according to epidemiological data on primary liver cancer collected by JECFA, an intake of 1.0 ng kg^−1^ b.w./day would increase the incidence of cancer in the liver at 0.013 cancers/year/100,000 inhabitants, so the EDI obtained is quite of concern to the health of consumers.

##### Zearalenone

**Figure 2:**
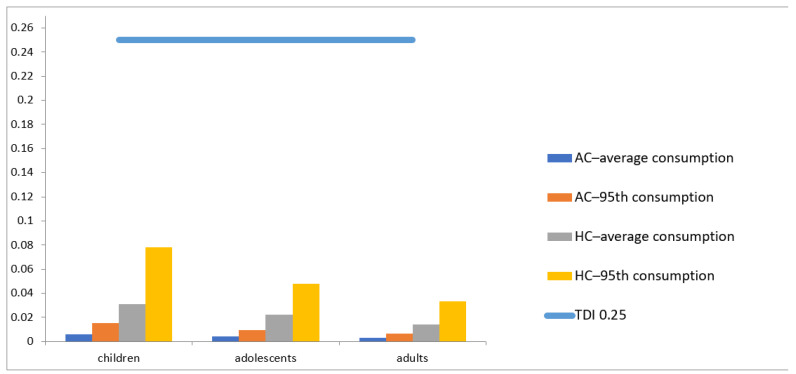
ZEN estimated daily intake (EDI) and risk assessment.

The current ZEN tolerable daily intake (TDI), 0.25 µg kg^−1^ b.w./day, established by the EFSA Panel for Contaminants in the Food Chain (CONTAM Panel) in 2011, is based on its oestrogenicity in pigs [73]. Regarding ZEN, as observed in Table 4 and Figure 2, the maximum EDI was 0.078 µg kg^−1^ b.w./day, a value obtained when considering the worst-case scenario (highest concentration) and the 95th consumption of children, with consequently higher risk values, 31.1%. Nonetheless, in every case, the EDI was far from the established TDI, ranging from 0.00275 µg kg^−1^ b.w./day and 0.078 µg kg^−1^ b.w./day, corresponding to a calculated risk of 1.10% and 31.1%, respectively.

When considering the average concentration (AC)/average consumption approach, lower EDI and risk values were obtained, as expected, ranging between 1.10 and 2.40%.

Again, in every approach considered, children presented higher risk values considering this TDI value. Accordingly, other authors described children as a susceptible group to most contaminants [15].

#### 2.2.2. Inorganic Arsenic

**Figure 3:**
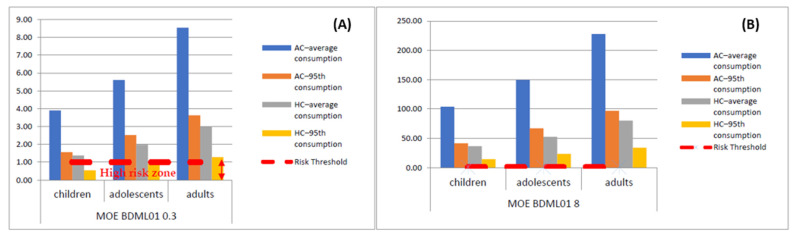
InAs risk assessment: (**A**) MOE using the BDML01 of 0.3 µg kg^−1^ b.w./day and (**B**) MOE using the BDML01 of 8 µg kg^−1^ b.w./day.

For every population in the study, different EDI assessments were attempted based on different scenarios, including the AC approach, the HC approach, and both combined, with the average and 95th percentile consumption [70]. As shown in Table 5 and in Figure 3, the maximum calculated EDI value was 0.5463 µg kg^−1^ b.w./day, obtained for children when the HC–95th consumption scenario was considered. When considering the AC–average consumption scenario, the EDI values were 0.0770, 0.0535, and 0.0351 µg kg^−1^ b.w./day for children, adolescents, and adults, respectively.

Risk assessment (Table 5 and Figure 3) was accomplished by comparison of the obtained EDI with the BMDL01 of 0.3 and 8 µg kg^−1^ b.w./day, established by the EFSA’s Panel on Contaminants in the Food Chain [74], obtaining the MOE. If MOE < 1, risk cannot be excluded. As expected, children were the population with higher risk values, once again following adolescents and adults. The ratio of BDML_01_0.3/EDI, using the AC-average consumption scenario, was 3.90, 5.61, and 8.54 for children, adolescents and adults, respectively. The MOE decreased when the 95th percentile was considered for values lower or close to 1:0.55, 0.89, and 1.28, for children, adolescents, and adults, respectively. Certainly, when considering the BMDL01 to be 8 µg kg^−1^ b.w./day, higher MOE values were obtained, ranging between 14.65 for children when considering the HC–95th consumption scenario and 227.61 for adults considering the AC–average consumption scenario.

Compared with other published data, other authors also found that toddlers and infants presented the highest dietary exposure to total As, 4.08 and 3.99 μg/day, respectively. However, in contrast with the other population groups, the major contributor was organic arsenic, and none of the population groups surpassed the lower limit of the BMDL01 range (0.3 μg kg^−1^ b.w./day) established by the EFSA in every exposure scenario considered (high, mean, and low) [61]. Other studies also reported that toddlers ingest large amounts of rice-based products, namely, cereals and rice drinks and present a higher risk of InAs intake; thus, a possible health risk should not be excluded. In numerous scenarios, InAs exposure was estimated to be above the EFSA’s lower BMDL01 of 0.3 µg kg^−1^b.w./day, but in no scenario, it was above the upper BMDL01 of 8 µg kg^−1^ b.w./day established by EFSA [62].

On the contrary, in Finland, others observed that the InAs exposure from the consumption of long grain rice and rice-based baby food in all age groups is close to the lowest BMDL0.1 value, considering conservative worst-case scenarios estimations, reaching a maximum of 0.67 μg kg^−1^ b.w./day for children [57].

## 3. Conclusions

This pilot survey showed that rice is widely contaminated with InAs and ZEN, with frequencies of contamination of 100% and 88.89%, respectively. Nevertheless, none of these contaminants surpassed the established MPL in the European Union. Conversely to the field mycotoxin, the surveyed storage mycotoxins surveyed were scarce. Indeed, OTA was not detected, whereas AFB1 was found in two single rice samples. It is noteworthy that the two AFB1-contaminated rice samples featured levels close to and even slightly higher than the MPL.

No correlation was found between the mycotoxin and InAs contamination of rice samples. Nevertheless, statistical analysis showed a significant difference regarding mycotoxin levels when comparing private and white-label samples (*p* = 0.0196). White-label samples presented higher values and thus presented a higher contribution to the exposure of consumers.

Furthermore, when brown and white rice were compared, a significant difference (*p* < 0.0001) was observed in the contamination by InAs. Brown rice featured higher values in both maximum and average levels. A similar tendency was observed for ZEN, although without statistical significance.

Regarding exposure and risk assessment, for all three mycotoxins and InAs, and in every scenario considered, children presented the higher EDIs and risk values. Current legislation does not establish maximum permitted levels of mycotoxins, specifically in rice. The results obtained in this work can thus contribute to awareness and science-driven policies aimed at public health protection.

## 4. Materials and Methods

### 4.1. Sampling

A total of 36 rice samples intended for human consumption were collected between November 2019 and February 2020. A total of 22 samples were kindly provided by Portuguese rice producers, while the remaining samples (*n* = 14) were commercially acquired as available for regular consumers from different Portuguese supermarkets. The latest included 8 private labels (produced for exclusive sale by a specific retailer) and 6 white labels (products distributed by the manufacturer to many suppliers, who then resell the product under their own brand).

Convenience sampling gathered different types of rice: white rice (*n* = 22), brown rice (*n* = 13), and wild rice (*n* = 1). Regarding grain length, 20 were long grain, and 16 were short grain rice. Concerning origin, most samples were produced in mainland Portugal (*n* = 27), while 9 samples were imported (Canada (*n* = 1), China (*n* = 1), India (*n* = 4), Thailand (*n* = 2), and Uruguay (*n* = 1)).

The information available on the labels was gathered. Samples were thoroughly minced to ensure homogenization and prevent the non-uniform growth of the mycotoxigenic fungi. Until analysis, samples were stored in the dark and at room temperature. All samples were analysed uncooked (raw) before their expiration date.

### 4.2. Experimental Procedures

#### 4.2.1. Mycotoxins

For the quantification of AFB1, an immunoenzymatic test in a competitive format was performed according to the enclosed instructions of the commercial test kit (RIDASCREEN Aflatoxin B1 30/15; Art. No. TR1211, R-Biopharm, Darmstadt, Germany).

For OTA quantification, the competitive enzyme immunoassay kit RIDASCREEN Ochratoxin A 30/15, R1312 (R-Biopharm, Darmstadt, Germany) was used following the manufacturer’s instructions.

FOR ZEN determination, the RIDASCREEN Zearalenon (Art. Nr.: R1401) kit was applied, following the instructions of the manufacturer.

The standard curves in the enzyme immunoassays, AFB1, OTA, and ZEN were obtained with the mean values of each of the six duplicated concentration levels: 0, 1, 5, 10, 20, and 50 μg L^−1^; 0, 0.03, 0.1, 0.3, 1, and 3 μg L^−1^; and 0, 0.05, 0.15, 0.45, 1.35, and 4.05 μg L^−1^ respectively. Mycotoxin quantification was achieved using the software RIDASOFT.Win.net. The calculation performed in double determinations used a cubic spline function. According to the manufacturer, the limit of detection of the enzyme immunoassays was 1 μg kg^−1^ for AFB1, 0.8 μg kg^−1^ for OTA, and 1.75 μg kg^−1^ for ZEN.

#### 4.2.2. Inorganic Arsenic

For InAs analysis, 5 g of each minced sample was vortexed and digested with 50 mL of 1% nitric acid (1%) for 10 min, followed by ultrasound extraction for 15 min and centrifugation at 2880 g for 15 min. After filtration of the supernatant with a 0.45 μm filter, the extracts were diluted with 0.5% HNO_3_ prior to analysis by inductively coupled plasma mass spectrometry (ICP-MS) XSERIES-2, ThermoUnican, with peak jumping acquisition mode, at 1370 W (*m*/*z* 75), using the following conditions: number of main runs—5; extraction lens potential—−106 V; RF forward power—1370 V; dwell time—0.02 s; sampling depth—108 mm; nebulizer gas—0.87 L min^−1^.

### 4.3. Analytical Performance

#### 4.3.1. Mycotoxins

Regarding the AFB1 standard curve, the mean coefficient of variation (CV) was 2%, with the highest value being 7%. As for OTA, the mean CV was 2.5%, with the highest value being 6%. For ZEN, an average CV of 5.2% was obtained, with a maximum of 6.3% and a minimum of 2.6%.

Considering the AFB1, OTA, and ZEN maximum permitted levels in cereals for human consumption, 2, 3 and 75 μg kg^−1^, respectively, it is perceived that the LODs of the ELISA’s applied methodologies are adequate [17,18].

#### 4.3.2. Inorganic Arsenic

The calibration curve was performed with the following standards: 0.2, 0.4, 0.8, 1.0, 2.0, and 5.0 μg L^−1^ of arsenic prepared in 0.5% HNO_3_. An internal standard of scandium (Sc) (*m*/*z* 45) was used at 25 μg L^−1^. The correlation coefficient obtained was 0.9999.

The LOD and LOQ were determined by taking 3.3 and 10 times the standard deviation, respectively, plus the mean obtained for 10 blanks. The LOD and the LOQ obtained were 3.3 μg kg^−1^ and 10 μg kg^−1^, respectively [75].

Accuracy, calculated using 5 recovery assays, was 99%, 114%, 106%, 111% and 89%, with a precision of 10.8% [75].

Compared with validation results reported by other ICP-MS studies in rice, the currently applied methodology satisfies. Other authors using similar methodologies obtained a LOD of 11 μg kg^−1^ and a LOQ of 38 μg kg^−1^, with an accuracy of 108.7% [56], a LOQ of 100 μg kg^−1^, and a recovery of 91.8% [14] (Table 1).

### 4.4. Statistical Analysis

Statistical analysis was achieved with GraphPad Prism (6.01, GraphPad Software, Inc., San Diego, CA, USA). To assess if the datasets were Gaussian-distributed, D’Agostino–Pearson normality, Shapiro–Wilk and KS normality tests were applied. Given that the datasets were not normally distributed, non-parametric tests were used. To compare InAs concentrations in rice of different origins, the Kruskal–Wallis test with Dunn’s post-test was applied. For other comparisons, the Mann–Whitney test was used. The statistical significance level was established at *p* < 0.05. Spearman’s r test was used to assess correlation.

### 4.5. Calculation of Estimated Daily Intake and Risk Assessment

The estimated daily intake (EDI) was calculated through a deterministic method (IPCS, 2009) using the equation:

EDI = (Ʃc) (CN^−1^ D^−1^ K^−1^)

where Ʃc is the sum of the compound in the analysed samples (µg kg^−1^), C is the mean annual intake estimated per person, N is the number of analysed samples, D corresponds to the number of days in one year, and K stands for the body weight (kg).

According to the last report of the “National Food Survey and Physical Activity, IAN-AF 2015–2016”, the adult population averaged 25.1 kg of rice ingested annually per capita. Regarding children and adolescents, the annual ingestion was 19.1 and 28.3 kg/year, respectively. Concerning the 95th percentile, the annual ingestion was 47.9, 62.8, and 59.0 kg/year for children, adolescents, and adults, respectively [70]. For intake estimation, both average and 95th percentile consumption for the different populations were considered.

The mean body weight taken into account for the Portuguese adult population was 69 kg [76]. For children (2–12 years) and adolescents (13–18 years), 24 and 51 kg were used, respectively [77].

For AFB1 exposure estimation, and given that the percentage of censored data (results reported under LOD) was above 50%, two different scenarios, as reported by the EFSA, were considered: (1) the lower-bound (LB) approach, implemented by replacing the results below the LOD with zero, and (2) the upper-bound (UB) approach, implemented by replacing the results below the LOD with the LOD value [78]. The AFB1 risk assessment was evaluated using the provisional maximum tolerable daily intake (PMTDI) values proposed by Kuiper-Goodman [71] of 1.0 ng kg^−1^ b.w./day for adults and children without hepatitis B and 0.4 ng kg^−1^ b.w./day for those with the hepatitis B virus since this is a genotoxic carcinogenic compound and, therefore, no official TDI is set. As such, the AFB1 risk was also characterized by calculating the margin of exposure (MOE) approach. The MOE corresponds to the ratio between the lower benchmark dose (BMDL) for the critical effect and the exposure dose. An increased MOE implies a smaller risk, and a value below 10,000 indicates a concern for human health [72]. The BMDL10 (benchmark dose lower confidence limit of 10%, which represents the lower bound of a 95% confidence interval on a BMD corresponding to a 10% tumour incidence) of 170 ng kg^−1^ b.w./day, set by EFSA, was applied for calculating the MOE MOE [79].

Regarding ZEN, two exposure scenarios were considered. In the first one, the average concentration (AC) of ZEN in rice was considered. In the second, the worst scenario was verified using the highest concentration (HC) found. The risk was calculated, in percentage, through the ratio between the obtained EDI and the current tolerable daily intake (TDI) of 0.25 µg kg^−1^ b.w./day [73]. The obtained risk should be interpreted as the higher the ratio, the higher the risk.

Concerning InAs risk assessment, the EFSA Panel on Contaminants in the Food Chain established a BMDL01 ranging between 0.3 and 8 µg kg^−1^ b.w./day for a higher risk of cancer of the lungs, skin and bladder, as well as skin lesions [74]. The MOE equals the ratio between the selected BMDL value and the calculated intake. When MOE < 1, risk cannot be excluded; however, there no clear guidelines exist to interpret MOE values > 1 [55]. Risk assessment was conducted for both BMDL01, bearing in mind two exposure scenarios. In the first one, the average concentration (AC) of InAs in rice was used. In the second, the worst scenario was verified using the highest concentration (HC) found.

## Figures and Tables

**Table 1:** Data from previous studies reported in the scientific literature on the occurrence of aflatoxin B1, ochratoxin A, and arsenic in rice.

Country	Sample Type	Methodology	LOQ (LOD) (µg kg^−1^)	N Samples	Frequency (%)	Levels (µg kg^−1^)			Reference
						Min.	Máx.	Average	
Aflatoxins									
Austria	Rice	SPE(IAC)-LC-FD	0.44 (0.1)	81	29.6	<LOQ	9.86	ns	[21]
Canada	Rice	LC-MS	0.05 (0.002)	200	49.5	1.44	7.14	0.18	[22]
China	Rice	LLME-LC-FD	0.03 (0.009)	370	63.5	0.030	20.0	0.60	[23]
China	Rice	ELISA/HPLC	(0.1)	29	100	0.1	1.4	0.5	[24]
China	Rice	SLE-LC-FD	(0.05)	37	97.3	21	30	0.88	[25]
Philippines	Rice	SPE(IAC)-LC-FD	(0.025)	78	95	ND	8.33	1.48	[26]
Iran	Rice	ELISA	ns	40	100	0.29	2.92	2.09	[27]
Iran	Rice	SPE(IAC)-LC-FD	(0.1)	71	83	ND	10	1.89	[28]
Malaysia	Rice products	ELISA	0.35 (0.2)	13	69.2	0.68	3.79	1.5	[29]
Pakistan	Rice	SPE(IAC)-LC-FD	0.20 (0.04)	208	35	ND	21.30	8.31	[30]
Pakistan	Brown rice	ELISA	(1.0)	120	73.3	1.24	11.68	3.70	[31]
Pakistan	Rice	SPE(IAC)-LC-FD	(0.5)	20	25	1.5 a	10.8 a	4.6 a	[32]
Tunisia	Rice	SPE(IAC)-LC-FD	0.1 (0.05)	11	0	ND	ND	ND	[33]
Turkey	Rice	ELISA	(1)	100	58	ND	21.4 a	ns	[34]
Vietnam	Rice	SLE-LC-FD	0.22 (0.07)	100	51	ND	29.8	3.31	[35]
Ochratoxin A									
Brazil	Rice	SPE(IAC)-LC-FD	(0.10)	165	28	ND	30.24	1.78	[36]
Canada	Rice	SPE(IAC)-LC-FD	0.2 (0.05)	100 99	43 1.01	ND	0.49	0.11 0.49	[22]
Chile	Rice	SPE-LC-FD	2.1 (0.6)	31	42	0	12.5	ns	[37]
China	Rice	SLE-LC-FD	0.3 (0.08)	370	4.9	0.3	3.2	0.85	[23]
Iran	Rice	ELISA	0.625	308	9.4	0.84	11.37	3.6 ± 2.66	[38]
Côte d’Ivoire	Rice	SPE-LC-FD	0.05 (0.01)	10	100.0	9.0	92.0	ns	[39]
Malaysia	Rice			20	0	NQ	NQ	NQ	[40]
Morocco	Rice	SPE(C8)-LC-FD	0.021	20	90.0	0.02 ± 0.01	32.4 ± 2.10	4.15 ± 1.45	[41]
Pakistan	White rice Brown rice	SPE(IAC)-LC-FD	0.18 (0.06)	34 28	29.4 46.4	ns ns	24.9 25.4	8.5 ± 0.6 7.84 ± 0.9	[30]
Portugal	Rice	SPE(IAC)-LC-FD	0.05	42	14.2	0.09	3.52	ns	[42]
Portugal and Spain	Organic rice Conventional rice	SPE(C8)-LC-FD	0.19 (0.05)	9 4	44.4 0	2.10 NQ	7.60 NQ	2.57 ± 3.43 NQ	[43]
Singapore	Rice	SLE-LC-MS-MS	0.4 (0.2)	190	0.5	46.5	46.5	46.5	[44]
South Korea	Rice	SPE(C18)-LC-FD	(1)	88	9.0	2.1	6.0	3.9	[45]
Spain	Rice	ASE-LC-FD	0.03 (0.01)	64	7.8	4.3	27.3	0.74	[46]
Tunisia	Rice	ELISA	(0.625)	16	25.0	0.8	2.3	1.4	[47]
Tunisia	Rice	SPE(IAC)-LC-FD	0.15 (0.05)	96	28	10	150	44	[48]
Turkey	Rice	ELISA	(0.025)	100	38.0	0.042	3.02	0.83	[49]
Vietnam	Rice	SLE-LC-FD	0.25 (0.08)	100	35.0	0.08	2.78	0.75	[35]
Zearalenone									
R. Korea	Rice	SLE-LC-FD	4	88	3.4	21.7	47.0	38.5	[45]
Côte d’Ivoire	Rice	ELISA	ns	10	100	50	200	95	[50]
Côte d’Ivoire	Rice	QuEChERS-UHPLC-MS-MS	5 (2.5)	9	21.05	<LOQ	7.5	6.6	[51]
Tunisia	Rice	ELISA	0.025	16	0	ND	ND	ND	[47]
Brazil	Rice	SPE (MycoSep)-LC-FD	(3.6)	166	29	ND	4872	143	[36]
Brazil	Rice	DSP-UHPLC-MS-MS	58.6 (29.3)	42	2.38	ns	ns	67	[52]
Pakistan	Rice	SPE(C18)-LC-MS-MS	13 (7)	180	15	ND	114	8.48	[15]
Vietnam	Rice	QuEChERS-LC-MS-MS	1.5 (0.5)	144	0	ND	ND	ND	[53]
Algeria	Rice	QuEChERS-UHPLC-MS-MS	8.4 (2.5)	30	20	8.6	15.5	9.9	[54]
Arsenic									
Belgium	White rice Brown rice Asian rice Wild/colored rice	MWE-LC-ICP-MS	2–4	30	100	ns ns ns ns	ns ns ns ns	80–245 119–243 19–147 40–141	[55]
Brazil	Polished Brown Parboiled	MWE-ICP-MS	38.0 (11.0) b	27 8 2	100 100 100	ns ns ns	ns ns ns	<38.0–245.0 101.0–660.0 61–80	[56]
Finland	Long grain rice	MWE-LC-ICP-MS	10 (5)	ns	*n* = 8	90	280	160	[57]
Portugal	White rice Brown rice	SLE-LC-ICP-MS	8 for As(III) 17 for As(V)	22 17	100 100	ns ns	ns ns	62.9–121.2 119–190	[58]
Slovenia	Rice Polished Brown	MWE-LC-HG-AFS	(1) for As(III) (2) for As(V)	50 40 10	ns ns ns	28.9 28.9 74.3	211 211 147.0	90.2 51.1–125 111.0	[59]
Spain	White rice Brown rice	SLE-FI-HG-AAS	(130)	39	100	ns ns	ns ns	85 144	[60]
Spain	Rice	SLE-LC-ICP-MS	ns	121	ns	47	190	101	[61]
Switzerland	White rice Brown rice	SLE-IC-ICP-MS	10.3 (3.44) b	27 4	100 100	5.6 117	188 172	94.0 152	[62]
Thailand	White rice Sticky rice	SLE-ICP-MS	100	96 63	ns ns	<100 <100	254.9 262.0	134.0 124.5	[14]
Thailand	Rice	MWE-ICP-MS	2.0 (0.98)	55	ns	67 b	402 b	110–240 b	[63]
United Kingdom	Total rice	SLE-LC-ICP-MS	nsgg	42	100	65	286	129	[6]
United States	White rice	SLE-ESI-IT-MS	ns	40	100	25	271	112	[64]

ND—not detected. NQ—not quantified. ns—not specified. SPE—Solid phase extraction. LC—Liquid chromatography. FD—Fluorescence detection. SLE—Solid-liquid extraction. ASE—Accelerated solvent extraction. LLME—Liquid–liquid microextraction. MWE—Microwave extraction. DSP—Dilute and shoot protocol. FI-HG-AAS—Flow injection–hydride generation–atomic absorption spectrometry. ESI-IT-MS—Ion-trap, electrospray, mass spectrometry. a—Total aflatoxin. b—Total arseniAs shown in Table 1, compared with previously published studies, the OTA incidence reported by other authors was higher, averaging between 0.11 μg kg^−1^ in Canada [22] and 44 μg kg^−1^ in Tunisia [48]. In Portugal, in 2005, 42 samples of Portuguese rice from different origins were analyzed, and none surpassed the maximum limit, with only 14% of the samples featuring detectable levels [42].

**Table 2:** Frequency (%) and InAs levels (µg kg^−1^) in the analyzed samples.

Rice	Frequency (%)	Levels (µg kg^−1^)		*p* Value
		Min.–Max.	Mean ± SD	
Total (*n* = 36)	100	>LOD–100.0	35.3 ± 28.2	-
Supermarket (*n* = 14)	100	>LOD–90.0	25.9 ± 26.9	0.0419
Producers (*n* = 22)	100	>LOD–100.0	41.2 ± 28.0	
White brand (*n* = 6)	100	>LOD–23.0	10.5 ± 7.1	0.0220
Private brand (*n* = 8)	100	>LOD–90.0	37.5 ± 30.9	
Long grain (*n* = 16)	100	>LOD–90.0	26.9 ± 26.1	0.0480
Short grain (*n* = 20)	100	>LOD–100.0	41.9 ± 28.6	
Brown rice (*n* = 14)	100	23.0–100.0	55.1 ± 27.7	<0.0001
White rice (*n* = 22)	100	>LOD–80.0	22.6 ± 20.5	
Portugal (*n* = 27)	100	>LOD–100.0	38.3 ± 26.1	0.0272
Abroad (*n* = 9)	100	>LOD–90.0	26.1 ± 33.9	

**Table 3:** AFB1 estimated daily intake (EDI) and risk assessment.

	EDI (ng kg^−1^ b.w./day)			EDI/PMTDI1.0 (%)			EDI/PMTDI 0.4 (%)			MOE		
	Children	Adolescents	Adults	Children	Adolescents	Adults	Children	Adolescents	Adults	Children	Adolescents	Adults
LB–average consumption	0.26	0.18	0.12	26.2	18.2	12.0	65.5	45.5	29.9	648.9	933.5	1420.8
LB–95th consumption	0.66	0.40	0.28	65.6	40.5	28.1	163.9	101.2	70.3	259.3	419.8	604.9
UB–average consumption	3.42	1.61	1.06	341.9	160.9	105.7	854.6	402.2	264.2	49.7	105.7	160.8
UB–95th consumption	5.79	2.64	2.48	579.0	264.4	248.3	1447.6	661.0	620.6	29.4	64.3	68.5

**Table 4:** ZEN estimated daily intake (EDI) and risk assessment.

	EDI (µg kg^−1^ b.w./day)			EDI/TDI (%)		
	Children	Adolescents	Adults	Children	Adolescents	Adults
LB–average consumption	0.00601	0.00418	0.00275	2.40	1.67	1.10
LB–95th consumption	0.015042	0.009293	0.006449	6.02	3.72	2.58
UB–average consumption	0.031	0.022	0.014	12.44	8.65	5.68
UB–95th consumption	0.078	0.048	0.033	31.1	19.2	13.3

**Table 5:** Inorganic arsenic estimated daily intake (EDI) and risk assessment.

	EDI (µg kg^−1^ b.w./day)			MOE BDML_01_ 0.3			MOE BDML_01_ 8		
	Children	Adolescents	Adults	Children	Adolescents	Adults	Children	Adolescents	Adults
AC–average consumption	0.0770	0.0535	0.0351	3.90	5.61	8.54	103.95	149.54	227.61
AC–95th consumption	0.1926	0.1190	0.0826	1.56	2.52	3.63	41.55	67.25	96.90
HC–average consumption	0.2183	0.1518	0.0997	1.37	1.98	3.01	36.64	52.71	80.23
HC–95th consumption	0.5463	0.3375	0.2342	0.55	0.89	1.28	14.65	23.71	34.16

## Data Availability

The data presented in this study are available on request from the corresponding author.
